# Ankyrin Repeat Domain 1: A Key Regulatory Factor and Emerging Therapeutic Target in Heart Disease

**DOI:** 10.31083/RCM48749

**Published:** 2026-07-20

**Authors:** Ziyuan Wang, Qiong Du, Shizhong Zhang

**Affiliations:** ^1^Hubei Key Laboratory of Tumor Microenvironment and Immunotherapy, China Three Gorges University, 443002 Yichang, Hubei, China; ^2^Department of Neurology, Yiling People’s Hospital of Yichang, 443002 Yichang, Hubei, China

**Keywords:** ankyrin repeat domain 1, cardiovascular disease, cardiac remodeling, cardiomyopathy, myocardial infarction, heart failure

## Abstract

Cardiovascular disease (CVD) remains the leading cause of death worldwide, highlighting the urgent need to identify effective therapeutic targets. Ankyrin repeat domain 1 (ANKRD1), also known as cardiac ankyrin repeat protein (CARP), is a member of the muscle ankyrin repeat protein (MARP) family. The unique multi-domain cooperative mechanism and dynamic nucleocytoplasmic shuttling associated with ANKRD1 confer increasingly significant roles in the pathogenesis and progression of CVD. This review aims to summarize the current understanding of the molecular structure of ANKRD1, elucidate the mechanisms underlying key cardiovascular pathological processes, including myocardial remodeling, cardiomyopathy, cardiac development, myocardial infarction, and heart failure, and comprehensively evaluate its potential as a therapeutic target. Furthermore, this review aims to provide new theoretical foundations and future research directions for the precision diagnosis and treatment of CVD.

## 1. Background

Cardiovascular disease (CVD) stands as the leading cause of morbidity and mortality worldwide, posing a severe challenge to public health systems while significantly increasing the economic burden on patients [1]. Current research projects that 17.5 million people die from CVD annually, accounting for approximately 31% of all deaths, with the number of cases projected to rise to 23.6 million by 2030 [2,3]. CVD encompasses diverse pathological conditions, including coronary artery disease (CAD), cardiomyopathy, and heart failure. Its pathogenesis and pathological progression are highly complex. Despite significant advances in existing research, current therapeutic approaches remain limited. Therefore, identifying CVD biomarkers and therapeutic-specific targets is crucial.

Ankyrin repeat domain 1, (ANKRD1), also known as cardiac ankyrin repeat protein (CARP), belongs to the muscle ankyrin repeat protein (MARP) family. Other members of this family include Ankyrin repeat domain protein 2 (ANKRD2) and diabetes related ankyrin repeat protein (DARP) [4,5]. Over the past few decades, significant advances have been made in understanding the ANKRD1 protein.

The discovery of ANKRD1 dates back to 1995, when Chu et al. [6] identified it as a novel cytokine-induced gene through differential screening of cDNA libraries prepared from human dermal microvascular endothelial cells stimulated with interleukin-1α (IL-1α) and tumor necrosis factor-α (TNF-α). They designated it C-193, the early name for *ANKRD1*. Subsequently, in 1997, Jeyaseelan et al. [7] identified this protein while investigating whether doxorubicin (DOX) interferes with cardiac-specific regulatory pathways. They observed that its mRNA levels exhibited high sensitivity to DOX, further revealing its association with cardiac function. That same year, Zou et al. [8] similarly identified this factor by screening a rat neonatal heart cDNA library using the Y-box binding protein 1 (YB-1). They confirmed its specific high expression in myocardial tissue, detectable as early as embryonic day 8.5 (E8.5), thereby establishing its identity as a nuclear transcription co-activator regulating cardiac gene expression.

Over the past few decades, significant progress has been made in the study of ANKRD1 in cardiovascular diseases. This review aims to outline the characteristics of ANKRD1 and its role in cardiovascular diseases, focusing on recent advances while identifying current limitations. It further discusses the potential of ANKRD1 as a cardiac target.

## 2. Structural Features of ANKRD1

The cDNA of *ANKRD1* spans 1901 base pairs and exists as a single copy on the long arm of human chromosome 10 (10q23.31). It encodes a protein composed of 319 amino acids with a theoretically predicted molecular weight of approximately 36 kDa. This protein exhibits high sequence conservation across mammals and features a complex domain architecture. Its domains include an anchor protein-like repeat sequence, a proline, glutamic acid, serine, and threonine (PEST)-rich region, an acidic amino acid cluster with nuclear localization signal function, phosphorylation sites, and a curled-coil domain. The synergistic interaction of these domains provides the molecular basis for its diverse functions [6,9].

Within its core structural domain, the C-terminus of ANKRD1 contains five tandemly arranged ankyrin repeat sequences [7]. Each ankyrin repeat is characterized by an L-shaped structure, which is formed by a basal β-hairpin and two antiparallel α-helices. Hydrophobic interactions stabilize the helical structure between repeat units, while a network of hydrogen bonds connects the β-hairpin regions, thereby establishing a stable spatial conformation [10,11,12].

The PEST sequence of ANKRD1 is located at amino acid residues 14–29 and 108–123. This sequence is a characteristic region that is rich in proline (P), glutamic acid (E), serine (S), and threonine (T). It functions as a signal tag for rapid intracellular protein degradation, and is a hallmark molecular feature of short-lived proteins. Typical short-lived proteins such as c-Myc, p53, and ornithine decarboxylase (ODC) all contain PEST sequences. The presence of PEST sequences in ANKRD1 suggests that ANKRD1 may undergo rapid intracellular degradation. This allows ANKRD1 to dynamically regulate its own protein levels in order to adapt to cellular physiological demands [13,14,15].

ANKRD1 contains a core nuclear localization signal (NLS) at its N-terminus (aa 71–74) and a bipartite NLS (aa 59–76) that includes the core NLS. This dual NLS structure enables ANKRD1 to shuttle from the cytoplasm to the nucleus [13]. In early studies, Dhyani et al. observed ANKRD1 in the cytoplasm through immunofluorescence experiments. Subsequent research further confirmed its presence within the nucleus [16,17]. ANKRD1 also has a predicted nuclear export signal [18], although its specific function remains unknown and require further investigation.

**Fig. 1.**
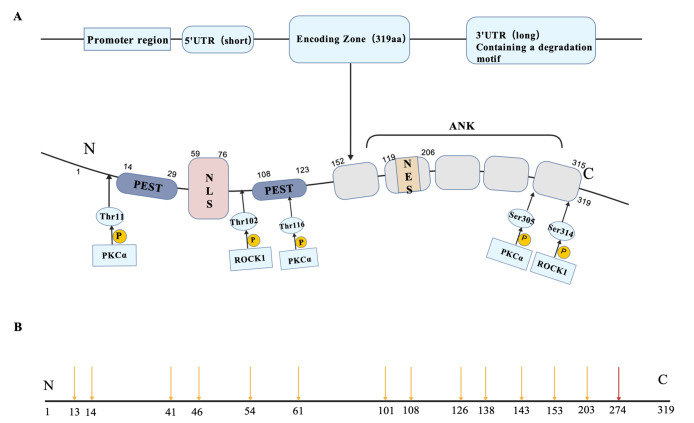
**Gene and protein structural features of ANKRD1 (Created with BioGDP.com) [26]**. (A) The *ANKRD1* gene consists of a short 5’ UTR, a coding region that encodes 319 amino acids, and a long 3’ UTR containing a degradation motif. Its promoter, located upstream of the 5’ UTR, contains multiple cis-acting elements. The coding region encompasses proline, glutamic acid, serine, and threonine (PEST) sequences, a nuclear localization signal (NLS), a nuclear export signal (NES) and ankyrin repeats. Several phosphorylation sites have been predicted and partially validated (Thr11-PKCα, Thr116-ROCK1, Ser314-ROCK1). These sites are primarily distributed flanking the ankyrin repeat domains and near clusters of basic amino acids, regulating the subcellular localization and signaling complex assembly of ANKRD1. (B) Potential acetylation sites (indicated in red) and ubiquitination sites (indicated in yellow) have been predicted using the PhosphoSitePlus database, suggesting complex post-translational regulation of ANKRD1 function. PKCα, protein kinase C; ROCK, Rho kinase.

Post-translational modifications (PTMs), as key mechanisms that regulate protein function, and also play a crucial role in the functional regulation of ANKRD1. Sequence analysis predicts that ANKRD1 has multiple potential PTM sites on, including phosphorylation, N-myristoylation, N-glycosylation, and amidation. Multiple phosphorylation sites, such as Thr11, Thr116, and Ser314, can be phosphorylated by protein kinase Cα (PKCα), casein kinase II (CKII), and Rho-associated coiled-coil containing protein kinase 1 (ROCK1), respectively. These sites are primarily distributed flanking the ankyrin repeat domains and near basic amino acid clusters [7]. Phosphorylation modifications may dynamically regulate biological functions by affecting transcriptional repression activity or altering protein-protein interaction interfaces [7,8,19]. N-myristoylation typically mediates protein binding to membranes and participates in subcellular transport and localization [20,21]. Prediction indicates two N-myristoylation sites in ANKRD1, suggesting its macroacetylation may be involved in non-canonical functional regulation [22]. N-glycosylation plays a crucial role in determining protein conformation and stability [23], while amidation is commonly found at the C-terminus of peptide hormones and affects the stability of protein interactions [22]. However, direct experimental validation of N-glycosylation and amidation modifications in ANKRD1 is currently lacking, and their functional significance remains to be further explored. Using the PhosphoSitePlus database, Mullenge et al. [24] predicted that ANKRD1 may also harbor acetylation sites and ubiquitinylation sites [25] (Fig. 1, Ref. [26]).

## 3. Intracellular Localization and Functional Regulation of ANKRD1

**Fig. 2.**
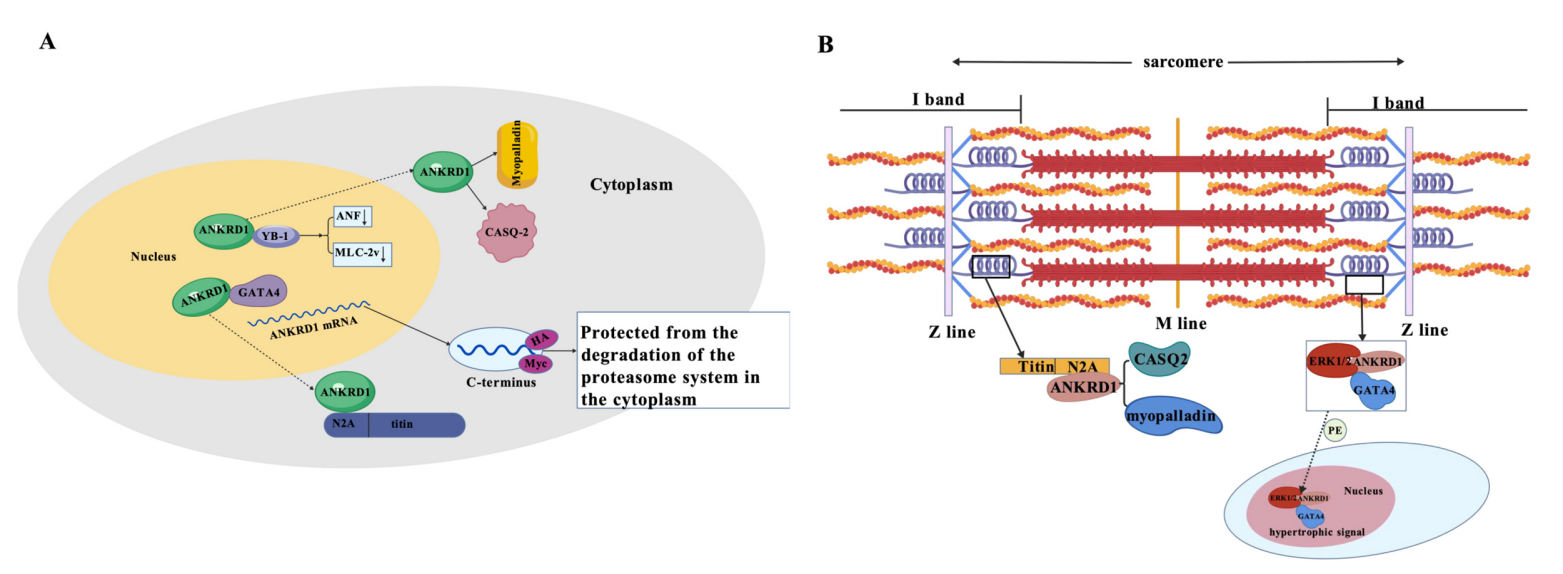
**Intracellular localization and functional regulation of ANKRD1 (created with BioGDP.com) [26]**. (A) As a regulatory factor, ANKRD1 interacts with various proteins such as YB-1 and GATA4 in the nucleus to modulate the expression of Atrial Natriuretic Factor (ANF) and Myosin Light Chain 2, Ventricular (MLC-2v) genes; In the cytoplasm, ANKRD1 binds to the N2A domain of titin and interacts with calsequestrin-2 (CASQ2) and myopalladin, contributing to the regulation of stretch sensing and sarcomere integrity; The C-terminal region of *ANKRD1* mRNA can be tagged with sequences such as HA or Myc, with its biological functions indicated. (B) ANKRD1 is specifically localized to the I-band of the sarcomere in cardiomyocytes, where it binds to the N2A domain of the giant sarcomeric protein titin to form a mechanosensory and transduction complex. This complex interacts with sarcomere-associated proteins such as CASQ2 and myopalladin to maintain sarcomeric structural integrity. Upon phenylephrine (PE) stimulation, the ANKRD1-titin complex recruits and phosphorylates extracellular signal-regulated kinases 1 and 2 (ERK1/2), which in turn phosphorylate GATA4, leading to the assembly of a signaling complex. Following nuclear translocation, this complex transmits cytoplasmic hypertrophic signals into the nucleus, where it regulates the transcriptional expression of downstream hypertrophy-related target genes, ultimately contributing to the development of pathological myocardial hypertrophy.

ANKRD1 is distributed across various cell types, including cardiomyocytes, inflammatory cells, epidermal cells, vascular endothelial cells, and smooth muscle cells [27]. It exhibits dual subcellular localization in both the nucleus and cytoplasm, with its function closely linked to its localization [5,28]. In the cytoplasm, it interacts with myosin filaments to maintain sarcomere structure. Cytoplasmic ANKRD1 also participates in regulating stretch sensing and myocardial contractility through interactions with myopalladin and cardiac calsequestrin-2 (CASQ2) [27]. Within muscle cells, it functions as a structural protein by binding to the N2A domain of the myosin giant protein titin, forming a signaling complex that participates in mechanical signal perception and transduction [5]. When cardiomyocytes are subjected to stimuli such as mechanical stretching, ANKRD1 in the cytoplasm is transported to the nucleus. Within the nucleus, ANKRD1 functions as a regulator of target gene expression. Acting as a transcriptional cofactor, ANKRD1 interacts with proteins such as YB-1 and modulates gene expression (e.g., by suppressing cardiac genes like Atrial Natriuretic Factor [ANF] and Myosin Light Chain 2, Ventricular [MLC-2v]) [8]. In endothelial and smooth muscle cells, ANKRD1 is activated by cytokines and enters the nucleus. By regulating gene expression programs, it enhances cellular migration and survival capabilities, thereby participating in tissue repair and angiogenesis [29]. Baumeister et al. [28] demonstrated post-transcriptional regulation of ANKRD1 in cells. By adding tags such as HA or Myc to the C-terminal region of ANKRD1, they observed that the protein escaped degradation by the cytoplasmic proteasome system. This suggests that the C-terminal structure serves as a degradation signal or susceptibility site, promoting protein retention and accumulation in the cytoplasm. Consequently, ANKRD1 functions as a sensitive signaling molecule capable of rapidly responding to changes in the intracellular and extracellular environments (Fig. 2, Ref. [26]).

Recent studies have also highlighted the pivotal role of the ubiquitin-proteasome pathway in regulating ANKRD1. This pathway maintains the intracellular homeostasis of ANKRD1 by modulating its degradation rate, with the 26S proteasome serving as the central catalytic role in this process [30].

## 4. The Role of ANKRD1 in Cardiovascular Diseases

Existing research indicates that studies on ANKRD1 function have primarily centered on cardiovascular and muscular disorders [19]. Although ANKRD1 exhibits distinct mechanisms across different cardiovascular diseases, its core functions can be categorized into the following types: transcriptional regulation, mechanical signal sensing, apoptosis regulation, and inflammatory response modulation. These mechanisms demonstrate both commonalities and specificities across various diseases, reflecting ANKRD1’s multifaceted role in cardiovascular physiology. In subsequent disease-specific subsections, we will analyze the mechanistic specificity of ANKRD1 within different pathological contexts based on this framework.

### 4.1 ANKRD1 and Cardiac Myocardial Remodeling

Cardiac remodeling refers to adaptive changes in the heart in response to pathological stimuli. Pathological myocardial remodeling is irreversible and constitutes a key pathological pathway in the progression of various cardiovascular diseases to heart failure. The triggers for myocardial remodeling signals are diverse, including myocardial infarction, ischemic/reperfusion injury following coronary artery revascularization and cardiopulmonary bypass surgery, pressure and volume overload, as well as neuroendocrine activation [31].

At the cellular level, myocardial remodeling primarily manifests as pathological alterations, including cardiomyocyte hypertrophy, myocardial fibrosis, and apoptosis. Initial myocardial hypertrophy represents an adaptive response to pressure or volume overload, sarcomeric protein mutations, and other stimuli. However, sustained hemodynamic overload on the cardiac wall triggers a cascade of reactions, ultimately progressing to myocardial fibrosis and heart failure [32]. Myocardial fibrosis is characterized by excessive deposition of extracellular matrix proteins. Excessive fibrosis induces myocardial scarring and organ dysfunction, ultimately leading to organ failure [31,33].

Furthermore, in myocardial remodeling, the inflammatory response is critical. Persistent activation of inflammatory cells leads to long-term myocardial damage, accelerating the progression of heart failure [32,34].

In studies of the molecular mechanisms underlying cardiac remodeling, the role of ANKRD1 has garnered significant attention. Through a series of animal experiments, the Aihara team first established ANKRD1 as a novel genetic marker for myocardial hypertrophy [35]. In models of pressure overload, hypertension, and Dahl salt-sensitive rats, ANKRD1 levels showed sustained elevation and exhibited a positive correlation with the degree of myocardial hypertrophy. Further mechanistic studies revealed that the p38/Rac1 signaling pathway significantly enhances *ANKRD1* transcription by specifically binding to the muscle-CAT (M-CAT) cis-acting element within its promoter region. This suggests ANKRD1 may participate in hypertrophic compensation by inhibiting myocardial protein synthesis. Its regulatory pattern as a tissue-specific nuclear factor offers new insights into understanding cardiac load adaptation responses.

However, findings regarding the specific role of ANKRD1 in cardiac remodeling vary across different research teams. Chen et al. [36] demonstrated that ANKRD1 overexpression promotes cardiac hypertrophy by activating the calmodulin-neurofilament phosphoprotein-NFAT signaling pathway. This mechanism may involve ANKRD1 translocating to the cytoplasm via calpain1, thereby exerting its hypertrophic effects. In contrast, Zhong et al. [37] proposed an alternative perspective. They found that ANKRD1 functions not merely as a transcriptional regulator but as a key scaffolding protein localized to the I-band of sarcomeres. It assembles a signaling complex containing extracellular signal-regulated kinases 1 and 2 (ERK1/2) and the cardiac core transcription factor GATA binding protein 4(GATA4) during sarcomere assembly. Under phenylephrine (PE) induction, ANKRD1 translocates to the nucleus, thereby transmitting hypertrophic signals into the nucleus. Knockdown of ANKRD1 not only significantly reduced ERK1/2 and GATA4 phosphorylation but also disrupted FHL-mediated ERK signaling within myofibrils, thereby attenuating myocardial hypertrophy (Fig. 2). Furthermore, Xie et al. [38] revealed that ANKRD1 directly binds to the promoter of the hypertrophy-associated gene Myosin heavy chain 7 (MYH7), activates its transcription, and induces embryonic myosin conversion (MYH6→MYH7), thereby promoting pathological myocardial hypertrophy.

Recent studies have also revealed that ANKRD1 plays a role in inflammation-related myocardial remodeling. Research using an experimental autoimmune myocarditis (EAM) model demonstrated that ANKRD1 deficiency significantly reduces the extent of myocardial remodeling and improves cardiac function by regulating the mitogen-activated protein kinase (MAPK)/activator protein-1 (AP-1) signaling pathway and mechanical sensing signaling pathways [39]. In the field of traditional Chinese medicine research, Meng et al. [40] discovered that Baoyuan Decoction (BYD), a classic qi-tonifying formula, alleviates myocardial hypertrophy and fibrosis by multi-target regulation of the ANKRD1-ERK/GATA4 signaling axis. Notably, Song et al. [41] observed in a model of myocardial hypertrophy induced by transverse aortic constriction (TAC) and β-adrenergic agonist (ISO) that ANKRD1 overexpression directly blocked the activation of ERK1/2 and TGF-β/Smad3 signaling pathways, significantly reducing ventricular hypertrophy and collagen deposition. Thus, the role of ANKRD1 in cardiac remodeling exhibits significant heterogeneity: some studies confirm its prohypertrophic effects (via ERK-GATA4 and calmodulin pathways), while others reveal its antihypertrophic function (by inhibiting the TGF-β/Smad3 pathway). These findings suggest that the differential expression of ANKRD1 in myocardial hypertrophy may stem from model dependence, subcellular localization, and interactions within signaling pathways. In Gq-coupled receptor agonist-induced hypertrophy (e.g., PE, Ang II), ANKRD1 acts as a scaffold protein within signaling complexes, serving as an essential factor for prohypertrophic responses. Conversely, in pressure overload-induced mechanical stress hypertrophy, its function can be compensated by MARP family members, and overexpression may even initiate negative feedback inhibition. This may result from dominant negative effects (interfering with MARP family member interactions) or off-target effects (abnormal binding to non-target proteins) induced by overexpression. Additionally, cytoplasmic/sarcomeric ANKRD1 promotes hypertrophy by assembling ERK-GATA4 signaling complexes, while nuclear ANKRD1 exerts antihypertrophic effects through direct regulation of gene transcription. Finally, physiologically expressed ANKRD1 serves as a normal response factor to mechanical stress; overexpression may reverse this function due to dominant negative effects or non-specific binding. This further demonstrates that ANKRD1 is a key mediator of myocardial hypertrophy under specific stimuli (Table 1, Ref. [35,37,39,40,41,42,43,44,45]).

**Table 1.** **The integrated regulatory network of ANKRD1 and major core signaling pathways**.

Signaling pathway	Related pathways	key molecules	Specific mechanism	Biological effects	Related diseases/models	References
GATA4-related pathways	Transcriptional Regulation of GATA4-ANKRD1	GATA4	GATA4 regulates ANKRD1 expression as an upstream transcription factor.	Maintain sarcomere integrity and inhibit apoptosis	Anthracyclin-induced cardiomyopathy	[42]
	As a scaffold protein, ANKRD1 assembles the ERK-GATA4 complex	ERK1/2, GATA4	ANKRD1 assembles the ERK1/2-GATA4 complex as a scaffold protein and transmits hypertrophic signals upon stimulation.	Promote myocardial hypertrophy	PE-induced myocardial hypertrophy	[37]
	ANKRD1 and GATA4 synergistically activate Bcl-2	GATA4, Bcl-2	ANKRD1 forms a complex with GATA4, which jointly binds to the Bcl-2 gene promoter to upregulate Bcl-2 expression.	Anti-apoptotic, reducing myocardial injury	I/R	[44]
	BYD regulates the ANKRD1-ERK/GATA4 axis	ERK1/2, GATA4	BYD suppresses the activation of ERK1/2 and GATA4 by regulating the signaling axis.	Anti-hypertrophic and anti-fibrotic effects	Hypertrophic cardiomyopathy	[40]
MAPK Family Pathways	ANKRD1 inhibits ERK1/2	ERK1/2	Overexpression of ANKRD1 directly inhibits the activation of ERK1/2.	Anti-Hypertrophic and Anti-Fibrotic Effects on Myocardium	TAC and ISO-induced myocardial hypertrophy	[41]
	ANKRD1 deficiency regulates MAPK/AP-1	MAPK, AP-1	ANKRD1 deficiency significantly attenuates cardiac remodeling by regulating the MAPK/AP-1 pathway.	Reduce myocardial inflammation and myocardial remodeling	Autoimmune Myocarditis	[39]
	p38/Rac1 activates ANKRD1 transcription	p38, Rac1	Activation of the p38/Rac1 signaling pathway binds to the cis-acting element of ANKRD1, enhancing its transcription.	Promote the expression of myocardial hypertrophy markers	Myocardial hypertrophy caused by excessive stress and hypertension	[35]
NF-κB pathway	ANKRD1- NF-κB	p50	Directly binds to the p50 subunit of NF-κB, inhibiting its transcriptional activity.	Anti-inflammatory	I/R	[45]
TGF-β/Smad pathway	ANKRD1 inhibits TGF-β/Smad3	TGF-β, Smad	Overexpression of ANKRD1 directly inhibits the activation of ERK1/2.	Anti-Hypertrophic and Anti-Fibrotic Effects on Myocardium	TAC and ISO-induced myocardial hypertrophy	[41]
	TGF-β/Smad activates ANKRD1 transcription	TGF-β, Smad	Following TGF-β/Smad activation, ANKRD1 gene transcription is induced via the CAGA element.	Inhibition of VSMC Proliferation	Atherosclerosis	[43]

BYD, baoyuan decoction; MAPK, mitogen-activated protein kinase; AP-1, activator protein-1; NF-κB, Nuclear Factor Kappa-B; TGF-β, transforming growth factor-β; ERK1/2, extracellular signal-regulated kinases 1 and 2; VSMC, vascular smooth muscle cell; CAGA, CAGA box; ISO, β-adrenergic agonist; TAC, transverse aortic constriction.

In summary, the mechanism of ANKRD1 in cardiac remodeling is complex, and further investigation is required to fully elucidate the specific mechanisms at play. However, it is undeniable that ANKRD1’s pivotal regulatory role in cardiac remodeling positions it as a promising therapeutic target. Future in-depth exploration of its mechanisms will provide novel strategies for treating diseases associated with cardiac remodeling.

### 4.2 ANKRD1 and Cardiomyopathy

Cardiomyopathy is a group of heart muscle diseases primarily characterized by structural and functional abnormalities of the heart. Based on morphological features, it can be classified into hypertrophic cardiomyopathy (HCM), dilated cardiomyopathy (DCM), and arrhythmogenic cardiomyopathy (AC) [46]. Existing research has confirmed that dysregulation of ANKRD1 expression is closely associated with the pathological processes of multiple cardiovascular diseases, including HCM, DCM, arrhythmogenic right ventricular cardiomyopathy (ARVC), and drug-induced cardiomyopathy. The molecular mechanisms involved are multifaceted, encompassing gene mutations, abnormal signaling pathways, and disrupted protein interactions.

#### 4.2.1 Dilated Cardiomyopathy

The pathological hallmark of DCM is left ventricular dilation and systolic dysfunction that occurs in the absence of abnormal cardiac workload, often progressing to heart failure in advanced stages [47]. Current standard treatments for DCM include conventional pharmacotherapy (e.g., ACEI/ARB, beta-blockers) and device-based therapies (implantable cardioverter-defibrillator [ICD], cardiac resynchronization therapy [CRT]). Despite ongoing efforts to optimize of treatment regimens, patient prognosis remains limited. This is primarily due to the lack of targeted therapies that address the core pathological mechanisms of the disease [48].

In molecular etiology research on DCM, the role of ANKRD1 has gradually been elucidated. Zolk et al. [49] analyzed left ventricular tissue from patients with end-stage heart failure due to DCM and found that ANKRD1 was specifically overexpressed in diseased myocardium, with expression intensity positively correlated with disease severity. Subsequently, in 2009, Moulik et al. [50] conducted gene sequencing in patients with familial and idiopathic DCM, confirming for the first time that *ANKRD1* is a disease-causing gene for DCM. They identified three missense mutations in patients: P105S, V107L, and M184I. Further yeast two-hybrid experiments revealed that the M184I mutation causes loss of ANKRD1’s binding capacity to key sarcomere components (Talin-1/FHL2), while the P105S mutation only affected binding to Talin-1. These mutations ultimately disrupted the expression of tension-responsive genes in cardiomyocytes (e.g., p53, TGFβ1 pathway abnormalities), impairing mechanical signaling in cardiomyocytes [50]. In the same year, Arimura et al. [51] identified an FHL2 variant in DCM patients that significantly reduced its binding capacity to the titin-N2B fragment, further substantiating the central role of abnormal mechanical stress signaling pathways in DCM pathogenesis. Concurrently, Duboscq-Bidot et al. [52] sequenced the ANKRD1 gene in 231 DCM patients, identifying five missense mutations: T116M, A276V, E57Q, R66Q, and L199R (Table 2, Ref. [50,51,52,53,54,55,56]).

**Table 2.** **Summary of ANKRD1 mutations associated with cardiovascular disease**.

Mutation	Model	Associated Disease	Function	Reference
*P105S*	Yeast two-hybrid system	DCM	Disrupts binding to Talin-1; impairs mechanical signaling in cardiomyocytes.	[50,51]
*V107L*	Yeast two-hybrid system	DCM	Identified as disease-causing.	[50,51]
*M184I*	Yeast two-hybrid system	DCM	Loss of binding to Talin-1 and FHL2; disrupts mechanical signaling.	[50,51]
*T116M*	Gene sequencing	DCM/TAPVR	Enhances protein stability and transcriptional repression of ANF; disrupts pulmonary vein development.	[52,55]
*A276V*	Gene sequencing	DCM	Impairs transcriptional repression function.	[52]
*E57Q*	Gene sequencing	DCM	Impairs transcriptional repression function.	[52]
*R66Q*	Gene sequencing	DCM	Impairs transcriptional repression function.	[52]
*S187F*	Exome sequencing technology	Cardiac Septal Defects	Augments transcriptional repression activity (downregulates ANF); impairs nuclear localization.	[56]
*Pro52Ala*	Protein-protein interaction assay	HCM	Enhances binding to Titin-N2A and Myopalladin; disrupts mechanical signaling.	[53,54]
*Thr123Met*	Protein-protein interaction assay/EHT	HCM	Enhances binding to Titin-N2A and Myopalladin; disrupts mechanical signaling; enhances contractile response in EHT models.	[53,54]
*Ile280Val*	Protein-protein interaction assay	HCM	Enhances binding to Titin-N2A and Myopalladin; disrupts mechanical signaling.	[53,54]
*L199R*	Gene sequencing	DCM	Impairs transcriptional repression function.	[52]

FHL2, 4-and-a-half LIM domains 2; TAPVR, total anomalous pulmonary venous return; HCM, hypertrophic cardiomyopathy.

Functional experiments confirmed these mutations impair ANKRD1’s transcriptional repression function, disrupting downstream target gene regulation and ultimately causing ventricular dilation and contractile dysfunction. This study first established *ANKRD1* missense mutations as novel genetic causes of dilated cardiomyopathy. These studies not only expand the spectrum of pathogenic genes for DCM but also underscore the critical role of the mechanical stress signaling pathway as a core pathomechanism in DCM, providing new targets for clinical genetic diagnosis.

Recent studies have further elucidated the role of ANKRD1 in dilated cardiomyopathy (DCM). Bogomolovas et al. [57] analyzed clinical samples and found that ANKRD1 expression levels may serve as a potential molecular biomarker for its complete molecular network driving disease onset and progression requires further investigation.

#### 4.2.2 Hypertrophic Cardiomyopathy

HCM, the most common hereditary cardiomyopathy, is a structural and functional myocardial disorder characterized by left ventricular (LV) a wall thickness ≥15 mm in any myocardial segment [58]. In 2009, Arimura’s team [53] first identified three *ANKRD1* missense mutations (Pro52Ala/Thr123Met/Ile280Val) through a cohort analysis of 384 HCM patients. Functional experiments confirmed that these mutations significantly enhance the binding capacity of the N2A domain of Titin and Myopalladin. This abnormally enhanced protein interaction disrupts mechanical signaling at the Z/I bands of sarcomeres, impairing normal myocardial contraction and relaxation, ultimately leading to HCM [53,54]. These findings established the critical role of ANKRD1 in maintaining cardiac functional integrity.

As a key responsive factor in HCM, ANKRD1 exhibits characteristic transcriptional upregulation upon activation by various pathological stimuli, making it a crucial molecular marker for cardiac hypertrophy. Across multidimensional experimental systems-including transverse aortic constriction (TAC)-induced pressure overload models, hypertension stimulation models, and neurohormone-mediated hypertrophy models like isoproterenol (ISO) and angiotensin II (AngII), *ANKRD1* consistently exhibits sustained upregulation [35,41]. This further confirms its intimate association with HCM pathogenesis. Crocini et al. [54] investigated the impact of HCM-associated mutations on contractile function using engineered heart tissue (EHT) models. They found that the T123Met mutation enhances myocardial contractile responses, expanding the spectrum of pathogenic mutations in HCM. More significantly, they observed that even when mutant ANKRD1 accumulated in the nucleus, the expression of classic hypertrophy-related genes (ANP, β-MHC, troponin, etc.) showed no significant alteration. This suggests that ANKRD1 mutations affect myocyte contractile function through non-transcriptional regulatory pathways, offering a new perspective for HCM pathogenesis research.

Although existing studies have clearly demonstrated that ANKRD1 plays a crucial role in the pathophysiology of HCM by regulating gene expression associated with myocardial function and structural integrity, and by interacting with key cardiac signaling pathways that control myocardial growth and stress responses [36,37], research by Bang et al. [59] presents a different perspective. In their TAC-induced mouse model, triple knockout of MARP family members resulted in cardiac hypertrophy responses identical to those of wild-type mice. This suggests that although ANKRD1 is induced during cardiac stress and disease, it is not indispensable for maintaining fundamental cardiac function or responding to acute stress loads. This discrepancy likely stems from compensatory mechanisms *in vivo*, as well as fundamental differences in pathogenic mechanisms between human disease mutations and gene knockout models. These conflicting findings imply that the role of ANKRD1 in HCM may be tissue-specific, developmentally regulated, or compensated by alternative pathways. Further investigation is needed to clarify its precise mechanisms, with future research potentially focusing on exploring ANKRD1’s role under alternative stress conditions or within specific cell types.

#### 4.2.3 Anthracycline-Induced Cardiomyopathy

Doxorubicin (DOX), a widely used chemotherapeutic agent, is effective against various malignancies such as breast cancer, leukemia, and sarcoma. However, its clinical utility is substantially limited by severe dose-dependent cardiotoxicity. The pathological features of DOX-induced cardiomyopathy include swelling of the sarcoplasmic reticulum and mitochondria, cytoplasmic vacuolation, as well as extensive sarcomeres disorganization and loss. Oxidative stress is recognized as a central mechanism underlying DOX cardiotoxicity, particularly given the relatively limited antioxidant capacity of cardiac tissue [60,61].

In studies on the molecular mechanisms of DOX-induced cardiomyopathy, the functional relationship between the transcription factor GATA4 and ANKRD1 has attracted considerable interest. As a zinc finger transcription factor of the GATA family, GATA4 enhances cardiac hypertrophy and promote cardiomyocyte survival by regulating the expression of downstream target genes [62]. DOX treatment has been shown to reduce of GATA4 expression in cardiomyocytes, an effect recognized as a key event of DOX cardiotoxicity. Moreover, maintaining GATA4 levels effectively suppresses DOX-induced cardiomyocyte apoptosis [63].

ANKRD1 was initially identified as the cardiac doxorubicin-responsive protein due to its significant transcriptional suppression upon doxorubicin stimulation [7]. Chen et al. [42] demonstrated that DOX significantly suppresses *ANKRD1* expression by inhibiting its promoter activity, leading to myocardial sarcomere disruption. Notably, overexpression of ANKRD1 alone was insufficient to rescue sarcomere damage, indicating that while its downregulation serves as a marker of myocardial injury, targeting ANKRD1 independently is insufficient to counteract DOX-induced cardiotoxicity. Further studies revealed that GATA4, an upstream regulator of ANKRD1 [42,64], partially restored ANKRD1 expression and alleviated sarcomere disorganization when overexpressed. However, this protective effect of GATA4 overexpression was completely abolished upon ANKRD1 knockdown, confirming a tight functional linkage between the two factors [42]. Collectively, these studies suggest that the GATA4/ANKRD1 signaling axis holds promise as a potential therapeutic target for DOX-induced cardiomyopathy, though its specific clinical translational value remains to be further validated.

### 4.3 ANKRD1 and Atherosclerosis

Atherosclerosis is a chronic lesion of large and medium-sized arteries, characterized by the progressive deposition of lipid plaques within the subendothelial layer. This pathological process serves as the common pathophysiological basis for coronary artery disease (CAD), peripheral artery disease (PAD), and cerebrovascular disease. In severe cases, arterial lumen narrowing can lead to restricted blood flow, leading to tissue hypoxic injury [65]. The pathological evolution of atherosclerosis is multistep process that begins with endothelial dysfunction and proceeds through lipid infiltration, inflammatory cell recruitment, and foam cell formation, culminating in the development of mature plaques. These plaques may eventually rupture, triggering acute cardiovascular events [66]. In studies on the pathological mechanisms of atherosclerosis, ANKRD1 has been shown to play multifaceted roles, participating in the fine-tuning of inflammatory responses and influencing the proliferation kinetics of vascular wall cells. De Waard et al. [67] analyzed human and mouse atherosclerotic specimens using immunohistochemistry and molecular biology techniques. They observed that ANKRD1 was specifically expressed in endothelial cells and intimal smooth muscle cells (SMCs) overlying plaques, but not in medial SMCs or macrophages. Further analysis using proliferation markers (osteopontin and PCNA) revealed that no colocalization occurred between ANKRD1 expression and these markers. This suggests ANKRD1 may mark a non-proliferative subtype or state of intimal SMCs. Notably, ANKRD1-positive SMCs exhibited mutually exclusive distribution with proliferative/activated SMC populations. Specifically, ANKRD1 expression increased in early-activated medial SMCs, whereas only a small fraction of late-quiescent medial SMCs retained ANKRD1 expression, indicating that ANKRD1 may be involved in regulating the transition of SMCs from an activated to a quiescent state. The study further revealed that activin A (a known factor that suppresses vascular lesions rich in SMCs and maintains the SMCs contractile phenotype [67]) can induce *ANKRD1* expression, accompanied by upregulation of SMCs contractile phenotype markers (such as SM α-actin and SM22α). This suggests that ANKRD1 overexpression may exert a protective role in vascular homeostasis by maintaining the SMCs’ contractile phenotype, thereby suppressing SMCs’ proliferation.

Furthermore, the research by Kanai et al. [43] confirmed that proinflammatory factors such as IL-1 and TNF-α, as well as activation of the transforming growth factor-β (TGF-β)/Smad signaling pathway, significantly upregulate *ANKRD1* expression. Mechanistic studies further revealed that ANKRD1 suppresses vascular smooth muscle cell (VSMC) proliferation via modulation of the p21-Rb pathway. Dysregulation of this pathway may contribute to excessive VSMC proliferation, thereby promoting atherosclerotic plaque formation and vascular stenosis. These findings imply that the ANKRD1-P21 axis may could represent a potential therapeutic target for inhibiting abnormal SMC proliferation within atherosclerotic plaques or vascular stenosis, offering a novel intervention strategy for atherosclerosis treatment. However, the *in vivo* regulatory mechanisms of this pathway and its clinical translational potential require further exploration.

Overall, ANKRD1 exerts a crucial regulatory role in the pathological progression of atherosclerosis by modulating SMCs’ phenotypic switching, suppressing inflammatory responses, and inhibiting VSMC proliferation. The equilibrium of its activity directly influences atherosclerotic progression, providing a novel intervention target and theoretical basis for preventing and treating this highly prevalent cardiovascular disease.

### 4.4 ANKRD1 and Myocardial Infarction and Ischemia-Reperfusion Injury

Myocardial infarction results from the acute obstruction of coronary blood flow, resulting in extensive necrosis of myocardial cells within the perfusion territory of the occluded vessel [68]. The current standard of care involves timely myocardial reperfusion achieved either by thrombolytic therapy or percutaneous coronary intervention (PCI). This approach is the most effective strategy to reduce infarct size, preserve left ventricular systolic function, and lower the incidence of heart failure. However, the restoration of blood flow can paradoxically exacerbate myocardial cell injury and induce cell death, known as myocardial ischemia/reperfusion injury (I/R injury) [69]. The pathogenesis of I/R involves multiple pathways, including reactive oxygen species (ROS) production [70], intracellular Ca^2+^ overload [71], rapid restoration of physiological pH [72], and activation of inflammatory responses [73]. These factors synergistically act through the opening of the mitochondrial permeability transition pore (mPTP) and induce excessive myocardial cell contraction, ultimately leading to myocardial cell death [74]. At present, no effective clinical interventions are available to specifically prevent myocardial reperfusion injury, underscoring the critical need to identify new therapeutic targets for this significant clinical problem.

Myocardial apoptosis is a key pathological process in ischemic heart disease, driven by dysregulated abnormal activation of oxidative stress, endoplasmic reticulum stress, and transcriptional regulatory networks. Among these, the endoplasmic reticulum stress-related transcription factor GADD153/CHOP is a member of the CCAAT/enhancer-binding protein (C/EBP) transcription factor family [75], and its overexpression can induce apoptosis [76]. In the context of myocardial ischemia/reperfusion (I/R) injury, Han et al. [77] employed an H9c2 cardiomyocyte hypoxia/reoxygenation (H/R) model and demonstrated that GADD153 binds directly to the ANKRD1 promoter region as a transcription factor, suppressing its transcriptional activity and thereby triggering the mitochondrial apoptosis pathway in cardiomyocytes. Further supporting this, Lee et al. [78] showed in both neonatal rat cardiomyocyte H/R and *in vivo* rat I/R injury models that activation of the AP-1/GADD153 pathway downregulates *ANKRD1* expression and ultimately promoting cardiomyocyte apoptosis. Collectively, these studies indicate that hypoxic stress suppresses ANKRD1 expression in cardiomyocytes via GADD153-mediated transcriptional suppression. The consequent downregulation of ANKRD1 is closely associated with increased cardiomyocyte apoptosis, underscoring its protective role in cardiomyocytes. Therefore, targeting the GADD153-ANKRD1 pathway may offer a novel therapeutic strategy to alleviate myocardial I/R injury.

Furthermore, Zhang et al. [44] identified another crucial mechanism through which ANKRD1 exerts its cardioprotective effects. In a myocardial ischemia/reperfusion model, they demonstrated that ANKRD1 associates with GATA4 to forms a complex that directly binds to the promoter of the anti-apoptotic protein gene Bcl-2. This promotes Bcl-2 expression and suppresses caspase-3 activation, ultimately reducing cardiomyocyte apoptosis. This discovery not only elucidates a broader role for ANKRD1 in regulating cell survival but also highlights the ANKRD1-GATA4-Bcl-2 signaling axis as a potential therapeutic target for mitigating myocardial I/R injury.

Recent studies have further elucidated the role of ANKRD1 in regulating inflammation during myocardial I/R injury. Through *in vitro* experiments, Liu et al. [45] demonstrated that ANKRD1 directly interacts with the P50 subunit of NF-κB, thereby leading to feedback inhibition of its transcriptional activity and fine-tuning of inflammatory balance, thereby uncovering a novel anti-inflammatory function of ANKRD1. In the context of traditional Chinese medicine (TCM) research, Xu et al. discovered that Storax, a classical Chinese herb, ameliorates cardiac function in rats with ISO-induced myocardial infarction by inhibiting the AT1R-ANKRD1-P53 signaling pathway. This intervention also attenuated cardiomyocyte apoptosis and reduced interstitial collagen fiber deposition, offering experimental support for TCM-based strategies targeting ANKRD1 signaling [79].

In summary, ANKRD1 participates in the pathological processes of myocardial infarction and I/R injury by regulating multiple signaling pathways, such as GADD153-ANKRD1, ANKRD1-GATA4-Bcl-2, and AT1R-ANKRD1-P53. Through its anti-apoptotic and anti-inflammatory functions, ANKRD1 significantly mitigates myocardial injury. These collective findings provide compelling experimental evidence for targeting ANKRD1 therapeutically; however, its clinical translational potential requires further validation.

### 4.5 ANKRD1 and Cardiac Development and Congenital Heart Disease

ANKRD1, a key regulator of cardiac development, exhibits mRNA expression in the cardiogenic plate as early as embryonic day 7.5 (before cardiogenesis) and continues to be detectable throughout adulthood. During cardiac development, ANKRD1 localizes specifically to cardiomyocytes, displaying a gradient distribution with atrial-ventricular segregation (higher in atria than in ventricles), followed by gradual attenuation in the adult heart [7]. Recent studies have further confirmed that ANKRD1 is highly expressed in the hearts of newborn mice, where it promotes Cyclin D1-dependent cardiomyocyte proliferation. Conversely, its downregulation in adulthood is associated with a loss of regenerative capacity in cardiomyocytes. Notably, myocardial-specific overexpression of ANKRD1 in adult mice significantly promotes cardiomyocyte proliferation in the infarct zone and improves cardiac function. These findings establish ANKRD1 as a core regulatory factor in cardiac development and regeneration [80], deepening our understanding of cardiac repair processes.

Congenital heart disease (CHD) encompasses a spectrum of disorders arising from structural malformations of the heart and major thoracic vessels. Its etiology is markedly multifactorial, involving both genetic factors (including chromosomal aberrations and mutations in key genes such as *TBX5* and *NKX2-5*) and intrauterine environmental exposures (e.g., maternal rubella infection, ethanol/ drug intake, gestational diabetes, and other teratogens), collectively forming a complex pathogenic network [81]. Within this genetic etiology research on CHDs, mutations and functional abnormalities in ANKRD1 have gradually become a focus of investigation.

Total anomalous pulmonary venous return (TAPVR) is a severe congenital heart defect characterized by the failure of the pulmonary venous plexus to connect properly to the left atrium. As a result, pulmonary venous blood drains into the right atrium or its tributaries, significantly impairing cardiopulmonary function [82]. Cinquetti et al. [55] first proposed *ANKRD1* as a candidate causative gene for TAPVR. They observed specific expression of ANKRD1 in the proximal embryonic pulmonary veins, suggesting its involvement in normal pulmonary vein development. Furthermore, they revealed that a missense mutation in *ANKRD1* (Thr116Met) enhances protein stability and transcriptional repression of ANF, disrupting the normal morphogenesis of pulmonary vein-left atrial development and ultimately leading to TAPVR. Subsequently, Yang et al. [56] identified another missense mutation (S187F) of *ANKRD1* via exome sequencing in patients with cardiac septal defects. Functional experiments demonstrated that this mutation augments the transcriptional repression activity of ANKRD1, downregulates ANF expression, and impair its nuclear localization capability, ultimately driving the pathogenesis of CHD. This study first established *ANKRD1* gene mutations as a novel genetic cause of cardiac septal defects.

Recent research has further advanced our understanding of ANKRD1’s role in congenital heart disease. Using a transgenic mouse model with myocardial-specific overexpression of ANKRD1, Piroddi et al. [83] demonstrated that ANKRD1 overexpression disrupts embryonic cardiac remodeling and postnatal sarcomere mechanical sensing. These alterations induce sinus-venous defects and progressive diastolic heart failure, establishing ANKRD1 as a molecular hub linking congenital structural heart abnormalities to acquired cardiomyopathy for the first time [83]. These findings not only underscore the imperative to continue exploring the genetic basis of CHD but also offer new perspectives for understanding its pathogenesis. A deeper investigation of ANKRD1 may holds promise for pioneering novel approaches to early detection and precision intervention in CHD.

### 4.6 ANKRD1 and Heart Failure

Heart failure (HF) is a chronic clinical syndrome characterized by the progressive decline of cardiac function. Its core pathophysiological alteration is the inability of cardiac output to meet the body’s metabolic and oxygen demands, which can ultimately lead to multi-organ failure and death. A wide spectrum of cardiovascular conditions can culminate in heart failure, including arrhythmias, cardiomyopathies, coronary artery disease, congenital heart disease, infections, hypertension, and valvular disease [84]. Although recent therapeutic advances in heart failure, such as SGLT2 inhibitors and ARNIs, have significantly improved clinical outcomes, approximately 20–30% of heart failure patients still die within five years of diagnosis [85]. Therefore, elucidating the molecular mechanisms driving heart failure and identifying novel therapeutic targets remains crucial for further improving patient prognosis.

In investigations of the molecular mechanisms of heart failure, abnormal expression and functional impairment of ANKRD1 have drawn considerable attention. Analysis of myocardial tissue from patients with heart failure due to dilated, ischemic, or arrhythmogenic right ventricular cardiomyopathy (ARVC) consistently show significantly elevated ANKRD1 levels in diseased myocardium, suggesting that its upregulation may contribute critically to the pathological progression of heart failure [49,57,86]. Further elucidating the regulatory mechanism, Xie et al. [38] reported that in both transverse aortic constriction (TAC) and DCM models, the nuclear-localized protein AGO2 is significantly upregulated and binds directly to the *ANKRD1* promoter region. This interaction transcriptionally activates *ANKRD1* expression, ultimately promoting the progression of heart failure. Importantly, the nuclear transport inhibitor ivermectin/ANPep effectively blocked ANKRD1 nuclear translocation and improved cardiac function. This work not only clarifies a regulatory pathway of ANKRD1 in heart failure but also provides experimental evidence for therapeutic strategies targeting ANKRD1 nuclear transport.

Cell death is a critical pathological process in the progression of heart failure. Shen et al. [87] demonstrated that overexpression of ANKRD1 in a transverse aortic constriction (TAC) model accelerates the deterioration of cardiac dysfunction and promotes cardiomyocyte apoptosis, whereas knockdown of ANKRD1 gene significantly mitigates the apoptotic process. Further studies in neonatal rat cardiomyocytes (NRCs) revealed that ANKRD1 overexpression synergistically exacerbates HF pathology by amplifying the cascade of Angiotensin II (AngII)-induced p53 activation, Bax mitochondrial translocation, and mitochondrial dysfunction. In a translational clinical study, Kempton et al. [88] analyzed samples from heart failure patients treated with left ventricular assist devices (LVADs), and identified ANKRD1 as a core mediator in the mechanical stress response of heart failure. They observed dynamic downregulation of ANKRD1 following LVAD intervention (validated in 6 patients), and further showed that its expression is regulated by the βII hemoglobin (cytoskeletal protein)-TGFβ pathway [88]. This insight offers a new perspective for understanding the mechanical stress adaptation mechanisms in heart failure.

Notably, Torrado et al. [89] reported that ANKRD1 exhibits differential expression between the left and right chambers in normal hearts. In a model of diastolic heart failure (DHF), ANKRD1 expression was significantly upregulated in the ventricles but downregulated in the atria. This pattern contrasts sharply with the global cardiac upregulation of ANKRD1 observed in systolic heart failure [89]. The divergence may arise from differing pathophysiological stresses, such as chronic ventricular stretch versus atrial systolic reserve depletion. Nevertheless, the precise mechanisms responsible for this chamber-specific remain incompletely elucidated and require further study.

Heart failure is closely associated with excessive activation of the neuroendocrine system, among which sympathetic nervous system hyperactivity represents a key pathological feature. In an isoproterenol (ISO)-induced rat model of myocardial hypertrophy and in neonatal rat cardiomyocytes (NRCs), Zolk et al. [90] demonstrated that the upregulating effect of ISO on ANKRD1 was completely blocked by either the PKA inhibitor Rp-cAMPS or the CaMK inhibitor KN-62, indicating that ISO regulates ANKRD1 expression through both PKA and CaMK signaling pathways. Further studies using an engineered heart tissue (EHT) model revealed that adenovirus-mediated ANKRD1 overexpression significantly attenuated the positive inotropic response of myocardial tissue to both calcium ion and ISO stimulation. This suggests that ANKRD1 elevation may contribute to the progression of heart failure by impairing contractile myocardial performance. These findings not only provides a molecular explanation for “β-adrenergic blunting” in heart failure but also establish a theoretical basis for targeting the ANKRD1 pathway to improve cardiac function (See Table 3 (Ref. [7,36,37,39,41,42,44,87,90]) for the functional mechanisms of ANKRD1 at the cellular level).

**Table 3.** **Functional mechanisms of ANKRD1 at the cellular level**.

Model type	Cell/Model description	Key findings	References
NRCs	Overexpression of ANKRD1	Enhances AngII-induced apoptosis (p53 pathway)	[87]
		Inhibits ERK1/2 and TGF-β/Smad3 activation, reducing hypertrophy and fibrosis	[41]
		Synergistically upregulates Bcl-2 anti-apoptosis with GATA4	[44]
NRCs	ANKRD1 Knockdown/Knockout	Attenuates PE-induced ERK1/2 and GATA4 phosphorylation, inhibiting hypertrophy	[37]
NMCs		Abnormal activation of the MAPK/AP-1 pathway alters cardiomyocyte apoptosis susceptibility	[39]
NRCs		Affects mechanical signaling	[42]
NRCs	Stretch Model	Inducing the translocation of ANKRD1 from the cytoplasm to the nucleus	[37]
		Mechanical stretching induces upregulation of ANKRD1 expression	[41]
NRCs	DOX-treated cardiomyocytes	Suppressing *ANKRD1 *expression	[7]
		Plays a crucial role in maintaining sarcomere integrity	[42]
	ISO-treated cardiomyocytes	Induces hypertrophic models and upregulates ANKRD1 via the PKA and CaMK pathways	[87,90]
	AngII-treated cardiomyocytes	Induction of hypertrophy and apoptosis	[36,87]

Studies have further demonstrated a positive correlation between ANKRD1 expression and the levels of established heart failure biomarkers, including proANP, NT-proBNP, and BNP [35,40,89]. Given the clinical utility of these natriuretic peptides in diagnosing and prognosticating heart failure, the observed correlation suggests that ANKRD1 may operate within shared stress-responsive pathways. This positions ANKRD1 as a potential novel molecular marker for tracking the development and progression of heart failure. However, its translation into clinical practice awaits validation through large-scale clinical studies.

In summary, ANKRD1 is a crucial regulatory role in the pathophysiology of heart failure by modulating cardiomyocyte apoptosis, mechanical stress adaptation, neuroendocrine signaling pathways, and natriuretic peptide-related pathways. Developing ANKRD1 inhibitors or modulating its associated signaling pathways holds promise as a potential future strategy for preventing and treating heart failure. However, its specific clinical translation requires further collaborative advancement through both basic and clinical research.

## 5. Conclusion and Perspectives

In summary, ANKRD1, as a key regulator of cardiac stress responses, exhibits significant expression alterations under pathological conditions such as cardiac stress load and cardiomyopathy. Its functions are highly diverse, encompassing transcriptional regulation, sarcomere structural maintenance, mechanical signal transduction, and apoptosis regulation. In the diagnosis and prognostic assessment of cardiovascular diseases, ANKRD1 has been identified as a potential novel diagnostic biomarker due to its cardiac-specific expression, dynamic regulation under pathological stimuli, and correlation with disease severity. Furthermore, its critical roles in myocardial remodeling, cardiomyopathy, myocardial infarction, and heart failure position it as an emerging therapeutic target.

Despite significant advances in basic research on ANKRD1 in cardiovascular disease, its clinical translation remains in the early stages. Currently, no targeted drugs for ANKRD1 or its key signaling axes (such as ANKRD1-GATA4, ANKRD1-P20) have entered clinical trial phases. Existing research is limited to observational, small-sample mechanism validation studies. These confirm the upregulation of ANKRD1 in human heart failure and its correlation with known biomarkers such as NT-proBNP and ANP. However, these findings lack prospective clinical trial data, leaving the prognostic or diagnostic value of ANKRD1 yet to be determined.

In the translational pathway, ANKRD1-targeting strategies also face multiple challenges: First, as a dual-localized nuclear-cytoplasmic protein, ANKRD1 exhibits significant subcellular localization dependence in its function. In particular, the dynamic regulatory signals governing nuclear-cytoplasmic shuttling and their physiological and pathological significance require further exploration. Second, ANKRD1 is also expressed in non-cardiac tissues (such as vascular smooth muscle cells, endothelial cells, and inflammatory cells). Systemic regulation of its expression may induce off-target effects, increasing therapeutic risks. Therefore, cardiac-specific delivery systems—such as adeno-associated virus (AAV) vectors and targeted nanoparticle carriers—are crucial for reducing adverse reactions, all of which require further research support.

Furthermore, patient stratification based on the *ANKRD1* gene remains unclear. Although *ANKRD1* mutation sites have been identified in cardiomyopathies such as DCM, the pathogenic mechanisms, functional impacts, and prevalence of these mutations across different populations have not been systematically evaluated, with a corresponding lack of clinical data. Therefore, integrating *ANKRD1* mutation screening with phenotype-related studies is crucial for patient stratification and personalized interventions.

Based on this, future research directions may focus on the following aspects: First, utilizing advanced technologies such as single-cell sequencing and proteomics to systematically analyze the expression patterns, protein interaction networks, and transcriptional activity of ANKRD1 in various cardiovascular diseases. This includes identifying drug modulators (e.g., small molecules, peptides, or oligonucleotides) to elucidate its core functional mechanisms and conduct systematic exploration. Second, develop animal models more closely resembling human disease (e.g., gene-edited large animal models) to rigorously validate the therapeutic efficacy of ANKRD1-targeted interventions, providing reliable evidence for clinical translation. Third, conduct multicenter, large-scale clinical studies to evaluate the clinical value of ANKRD1 as a diagnostic and prognostic biomarker for cardiovascular diseases, establish standardized detection platforms, and simultaneously explore the feasibility of novel ANKRD1-based therapeutic strategies (e.g., gene therapy, targeted drug development, local drug injection). Through sustained and in-depth research, we aim to further elucidate the mechanisms of ANKRD1 in cardiovascular diseases, advance its clinical application in disease diagnosis and treatment, and provide new breakthroughs for improving the prognosis of cardiovascular disease patients.
